# Relationship between overactive bladder and irritable bowel syndrome: a large-scale internet survey in Japan using the overactive bladder symptom score and Rome III criteria

**DOI:** 10.1111/j.1464-410X.2012.11591.x

**Published:** 2012-10-26

**Authors:** Seiji Matsumoto, Kazumi Hashizume, Naoki Wada, Jyunichi Hori, Gaku Tamaki, Masafumi Kita, Tatsuya Iwata, Hidehiro Kakizaki

**Affiliations:** Asahikawa Medical University, Renal and Urological SurgeryAsahikawa, Hokkaidou, Japan

**Keywords:** epidemiology, internet surveillance, overactive bladder, irritable bowel syndrome

## Abstract

**What's known on the subject? and What does the study add?:**

- There is known to be an association between overactive bladder (OAB) and irritable bowel syndrome (IBS).
- The study investigates the association between OAB and IBS using an internet-based survey in Japan. It is the first to investigate the prevalence and severity of OAB in the general population using the OAB symptom score questionnaire.

**Objective:**

- To investigate the association between overactive bladder (OAB) and irritable bowel syndrome (IBS) by using an internet-based survey in Japan.

**Subjects and Methods:**

- Questionnaires were sent via the internet to Japanese adults.
- The overactive bladder symptom score was used for screening OAB, and the Japanese version of the Rome III criteria for the diagnosis of IBS was used for screening this syndrome.

**Results:**

- The overall prevalence of OAB and IBS was 9.3% and 21.2%, respectively.
- Among the subjects with OAB, 33.3% had concurrent IBS.
- The prevalence of OAB among men was 9.7% and among women it was 8.9%, while 18.6% of men and 23.9% of women had IBS.
- Concurrent IBS was noted in 32.0% of men and 34.8% of women with OAB.

**Conclusion:**

- Taking into account a high rate of concurrent IBS in patients with OAB, it seems to be important for physicians to assess the defaecation habits of patients when diagnosing and treating OAB.

## Introduction

Overactive bladder (OAB) is characterized by symptoms of urgency, with or without urgency incontinence, usually with urinary frequency and nocturia in the absence of confirmed infection or other obvious pathologies 1. OAB is very common in the general population. Its prevalence according to a study in six European countries 2 was 16.6% among people aged ≥40 years and, according to a large-scale telephone survey conducted in the USA 3, it was 16.5% among those >18 years. Homma et al. 4estimated the overall prevalence of OAB in Japan to be 12.4%, with a prevalence of 14% among men and 11% among women aged ≥40 years, in their study using mailed self-administered questionnaires.

Irritable bowel syndrome (IBS) is a functional gastrointestinal disorder characterized by gastrointestinal symptoms such as abdominal pain or discomfort and alteration of bowel habits, despite the absence of an organic disease 5,6. IBS is very common in the general population of Europe and North America, with a reported prevalence of 10–20% 7–9. Miwa 10reported that the prevalence of IBS in Japan was 9.8% according to the Rome II criteria and 13.1% according to the Rome III criteria, using an internet survey.

Overactive bladder and IBS, both of which negatively affect quality of life, are characterized pathologically by overactivity (irritability) of the bladder and bowel, respectively, and previous studies have shown that they frequently occur concurrently 11–15. IBS is more prevalent in female patients with interstitial cystitis/painful bladder syndrome than in asymptomatic control subjects 16,17. Rat experiments have demonstrated hyperaesthesia of the bladder in a colitis-associated colon-hyperalgesia model 18 and colon hyperalgesia in a cyclophosphamide-induced cystitis model 19. In addition, the bladder and large intestine are reported to share, in part, common afferent nerve projections, suggesting the presence of neural cross-talk between these two organs 20,21. This has raised the possibility that OAB and IBS may share common pathological features. In the present study, we investigated the concomitant occurrence of OAB and IBS among Japanese adults through an internet survey on urine storage symptoms and defaecation habits.

## Subjects and Methods

In cooperation with the Social Survey Research Information Co, Ltd. (Shinjuku, Tokyo) that registered about 500 000 general consumers in Japan, 10 000 general consumers were selected as subjects using the stratified random sampling method. All subjects were at least 20 years of age and were stratified into five age groups (20–29, 30–39, 40–49, 50–59s and ≥60 years). The subjects included 5000 men and 5000 women, with 1000 in each age group. They were surveyed using the internet from 14 September 2010 to 15 September 2010. The subjects answered the Rome III criteria questionnaire at first and then the OAB symptom score (OABSS) questionnaire. The study was approved by the Asahikawa Medical University Ethical Committee.

The OABSS was developed by Homma et al. 22to detect OAB and assess its severity. The OABSS is a symptom assessment tool designed to combine OAB symptoms into a single score. It consists of four questions on symptoms: daytime frequency (Q1), night-time frequency (Q2), urgency (Q3), and urgency incontinence (Q4). Patients are asked to rate their symptom severity on a scale with a maximum (worst) score of 2, 3, 5 and 5, respectively. According to the clinical guidelines for OAB 23, OAB was defined as urinary urgency once a week or more (Q2 ≥ 2) and total score of OABSS ≥ 3. Total score of OABSS ranges from 0 to 15, with higher scores indicating increasing symptom severity (≥5, mild; 6–11, moderate; ≥12, severe) 23.

The Rome criteria represent a system developed to classify functional gastrointestinal disorders, i.e. disorders of the digestive system in which symptoms cannot be explained by the presence of structure or tissue abnormalities, on the basis of clinical symptoms. Examples include IBS, functional dyspepsia, functional constipation, and functional heartburn 24. IBS was defined using the IBS module (Appendix 1) of the Rome III criteria of a validated Japanese questionnaire 24–26., IBS was diagnosed when the module was applied to the conditions from Q1 to Q8. In addition, IBS subtypes (IBS-diarrhoea [IBS-D], IBS-mixed [IBS-M], IBS-constipation [IBS-C], and others/IBS-unsubtyped [IBS-U]) were determined according to the frequency of Q9 and Q10.

## Results

**Fig. 1:**
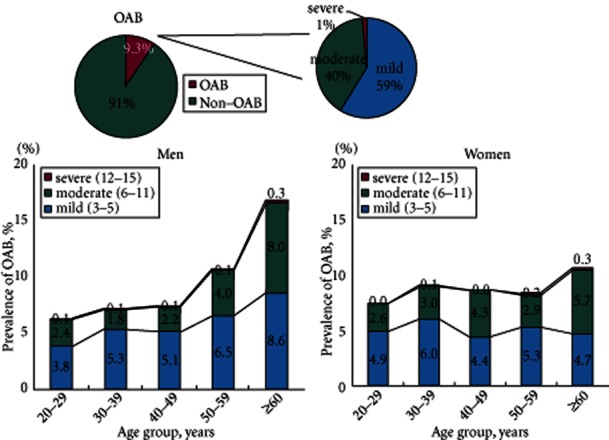
*Th*e prevalence of OAB, stratified by severity, gender and age, according to OABSS.

Overall OAB prevalence according to the OABSS was 9.3% (Fig. 1). The prevalence of OAB in men and women was 9.7% and 8.9%, respectively. According to the total OABSS, the severity of OAB was classified as mild in 59% of the subjects, moderate in 40% and severe in 1% (Fig. 1).

**Fig. 2:**
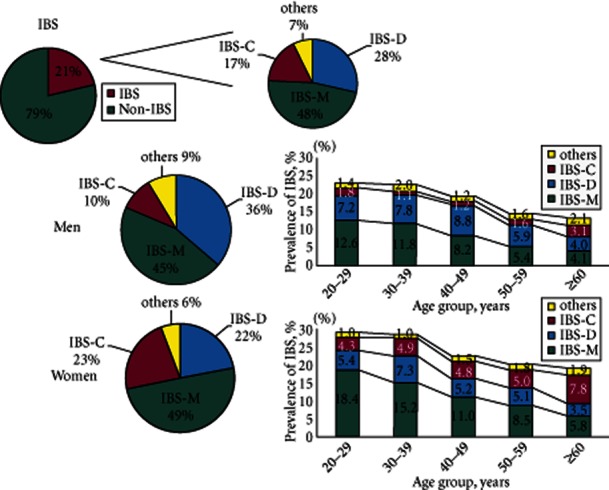
The prevalence of IBS, stratified by subtype, gender and age according to the IBS module of the Rome III criteria.

Overall IBS prevalence, according to the Rome III criteria, was 21.2% (Fig. 2). The prevalence of IBS in men and women was 18.6% and 23.9%, respectively. Among the subjects who met the Rome III criteria, 28%, 48% and 17% were categorized as having IBS-D, IBS-M and IBS-C, respectively (Fig. 2). In men, IBS-M was most common (45%), followed by IBS-D (28%) and IBS-C (10%), while in women IBS-M was most common (49%), followed by IBS-C (23%) and IBS-D (22%) with a similar frequency in the latter two subtypes.

**Fig. 3:**
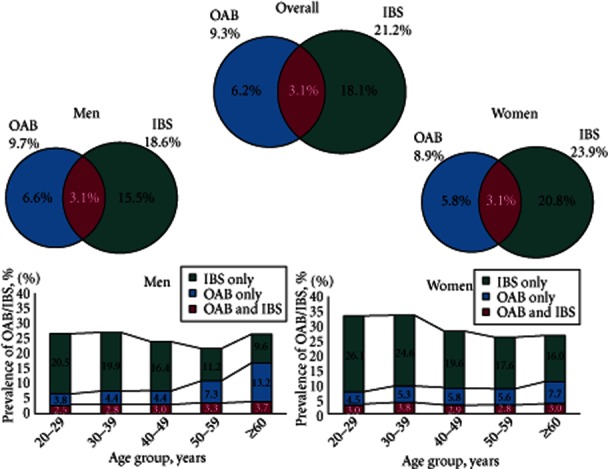
The overall prevalence of OAB and IBS, stratified by gender and age.

Overall, 33.3% of the patients with OAB had concurrent IBS (Fig. 3). Of the men with OAB, 32.0% had concurrent IBS and of the women with OAB, 34.8% had concurrent IBS. The prevalence of OAB increased in the age groups 50–59 and ≥60 years, while the prevalence of IBS decreased with age, especially in men. The prevalence of association of OAB and IBS was stable at around 3% of all five age groups. Stratified by severity of OAB, IBS was noted in 33.3, 32.8 and 38.5% of patients with mild, moderate and severe OAB, respectively. The prevalence of IBS was 33.3% in the subjects with OAB and 20.0% in the subjects without OAB (Fig. 3). The prevalence of OAB was 14.6% in the subjects with IBS and 7.9% in the subjects without IBS (Fig. 3). There was a significant difference between the prevalence of OAB and IBS (*P* < 0.001; chi-squared test).

## Discussion

We conducted a large-scale, internet-based epidemiological study to investigate the association between OAB and IBS among 10 000 general consumers across Japan. OAB and IBS were diagnosed using the OABSS and IBS module of the Rome III criteria. Because the study subjects included only those registered on the internet, a limitation of this survey was that doctors and other medical professionals were unable to assess them directly. Furthermore, some of the general problems encountered when using internet surveys, as compared with conventional interview surveys, are that respondents may pretend to be someone they are not and the selection of respondents is biased. For the present study, we took some measures to prevent such problems. We obtained personal information about the respondents and adopted a stratified random sampling method by gender and age. Consequently, as many as 10 000 consumers nationwide were enrolled into the study, with an equal number (1000) of subjects in each age group, so we consider the present data on the prevalence of OAB and IBS to be accurate. We found the prevalence of OAB increased with age, while the prevalence of IBS decreased with age. In fact, the prevalence of OAB and IBS in the present study is consistent with previous epidemiological studies in Japan 4,10; however, only 1% of the patients with OAB were classified as having severe OAB in the present study. We used severity classification criteria proposed by the clinical guidelines for OAB in Japan 23. This severity classification using the OABSS has never been compared with different questionnaires. The previous epidemiological study on OAB in Japan 4 was conducted before the development of the OABSS. The OABSS is the symptom assessment tool that is regarded as useful for clinical practice and research 22. The present epidemiological survey is the first to investigate the prevalence and severity of OAB in general population using the OABSS. Different questionnaires and severity criteria might result in different results. Future study is warranted to compare the prevalence and severity of OAB between countries using the OABSS.

Interestingly, the prevalence of association of OAB and IBS was stable at ∼3% of all five age groups, while the prevalence of OAB increased with age; therefore, the prevalence of concomitant IBS is higher in younger subjects with OAB than those aged 50–59 or ≥60 years.

Overactive bladder and IBS are pathologically characterized by overactivity (irritability) of the bladder and bowel, respectively, and previous studies have shown that they frequently occur concurrently 11–15. This has raised the possibility that OAB and IBS may share common pathological features; however, the pathophysiology of OAB and LUTS in patients with IBS is not well understood 11,12,15. IBS is more prevalent in female patients with interstitial cystitis/painful bladder syndrome 16,17. As mentioned above, rat experiments have also shown that hyperesthesia of the bladder can be found in a colitis-associated colon-hyperalgesia model and that colon hyperalgesia can be found in a cyclophosphamide-induced cystitis model 8,19, and the possibility of a neural cross-talk between the bladder and large intestine has been suggested 20,21. The potential link between LUTS and IBS may involve serotonin receptors, because serotonin is one of the key neurotransmitters within the urinary and gastrointestinal tract 27–29. Indeed, serotonin-related drugs have been studied for the treatment of OAB and IBS 27–30. Central nervous system communication with the urinary and gastrointestinal tract is mediated through the parasympathetic and sympathetic pathways. Patients with OAB and IBS have been shown to have autonomic dysfunctions. Further insight into the pathophysiology of OAB and IBS may lead to the development of more effective treatments for OAB and IBS.

In conclusion, OAB and IBS are pathologically characterized by overactivity (irritability) of the bladder and bowel, respectively. Although their prevalence rates differ depending on age and gender, the present survey showed that these diseases occurred concurrently in some cases and that OAB was complicated by IBS, particularly in about one-third of OAB patients. On the basis of these results, it would seem important to assess the defaecation habits of patients when diagnosing and treating OAB, especially younger patients with OAB patients. Because the present study focused only on OAB (storage symptoms) and IBS, further studies need to be conducted to examine the relationship between IBS and LUTS. It is also necessary to elucidate what impact IBS has on treatment outcome of patients with OAB.

## Abbreviations

OAB: overactive bladder
IBS: irritable bowel syndrome
OABSS: OAB symptom score
IBS-D: IBS-diarrhoea
IBS-M: IBS-mixed
IBS-C: IBS-constipation
IBS-U: IBS-unsubtyped

## Supporting Information

Appendix 1: IBS module.
