# Supplementary material for: Relationship between overactive bladder and irritable bowel syndrome: a large-scale internet survey in Japan using the overactive bladder symptom score and Rome III criteria
Source: BJU Int. 2012 Oct 26;111(4):647–52. doi: 10.1111/j.1464-410X.2012.11591.x (PMC3654175; doi:10.1111/j.1464-410X.2012.11591.x)
Supplement: Supplementary file 1 [file bju0111-0647-SD1.doc]

**Appendix 1: IBS Module**

**Diagnostic Criteria***

Recent abdominal pain or discomfort** at least 3 days/month in last 3 months associated with two or more of criteria #1 - #3 below.

***Pain or discomfort at least 2-3 days/month (question 1>2)***

***For women, does pain occur only during menstrual bleeding? (question 2=0 or 2)***

1. Improvement with defecation

***Pain or discomfort gets better after BM at least sometimes (question 4>0)***

1. Onset associated with a change in frequency of stool

***Onset of pain or discomfort associated with more stool at least sometimes (question 5>0), OR***

***Onset of pain or discomfort associated with fewer stool at least sometimes (question 6>0)***

1. Onset associated with a change in form (appearance) of stool

***Onset of pain or discomfort associated with looser stool at least sometimes (question 7>0), OR***

***Onset of pain or discomfort associated with harder stool at least sometimes (question 8>0)***

* Criteria fulfilled for at least 3 months with symptom onset at least 6 months prior to diagnosis

***Yes. (question 3=1)***

*** “Discomfort” means an uncomfortable sensation not described as pain.*

***In pathophysiology research and clinical trials, a pain/discomfort frequency of at least two days a week is recommended for subject eligibility.***

***Pain or discomfort more than one day per week (question 1>4)***

**Criteria for IBS-C:** *(question 9>0) and (question 10=0)*

**Criteria for IBS-D:** *(question 9=0) and (question 10>0)*

**Criteria for IBS-M:** *(question 9>0) and (question 10>0)*

**Criteria for IBS-U:** *(question 9=0) and (question 10=0)*
